# Supplementary material for: Mosaic deletion patterns of the human antibody heavy chain gene locus shown by Bayesian haplotyping
Source: Nat Commun. 2019 Feb 7;10:628. doi: 10.1038/s41467-019-08489-3 (PMC6367474; doi:10.1038/s41467-019-08489-3)
Supplement: Supplementary file 1 — Supplementary Information [file 41467_2019_8489_MOESM1_ESM.pdf]

Mosaic deletion patterns of the human antibody heavy chain gene locus shown by Bayesian haplotyping

Moriah Gidoni[1], Omri Snir[2], Ayelet Peres[1], Pazit Polak[1], Ida Lindeman[2], Ivana Mikocziova[2], Vikas Kumar Sarna[2], Knut E. A. Lundin[2], Christopher Clouser[3], Francois Vigneault[3], Andrew M. Collins[4], Ludvig M. Sollid[2], and Gur Yaari[1]

[1]Faculty of Engineering, Bar Ilan University, Ramat Gan 5290002, Israel

[2]KG Jebsen Centre for Coeliac Disease Research and Department of Immunology, University of Oslo and Oslo University Hospital, 0372 Oslo, Norway

[3]AbVitro, Inc., Boston, MA, USA

[4]School of Biotechnology and Biomolecular Sciences, University of NSW, Kensington, Sydney, NSW 2052 Australia

Supplementary material

Supplementary Figures

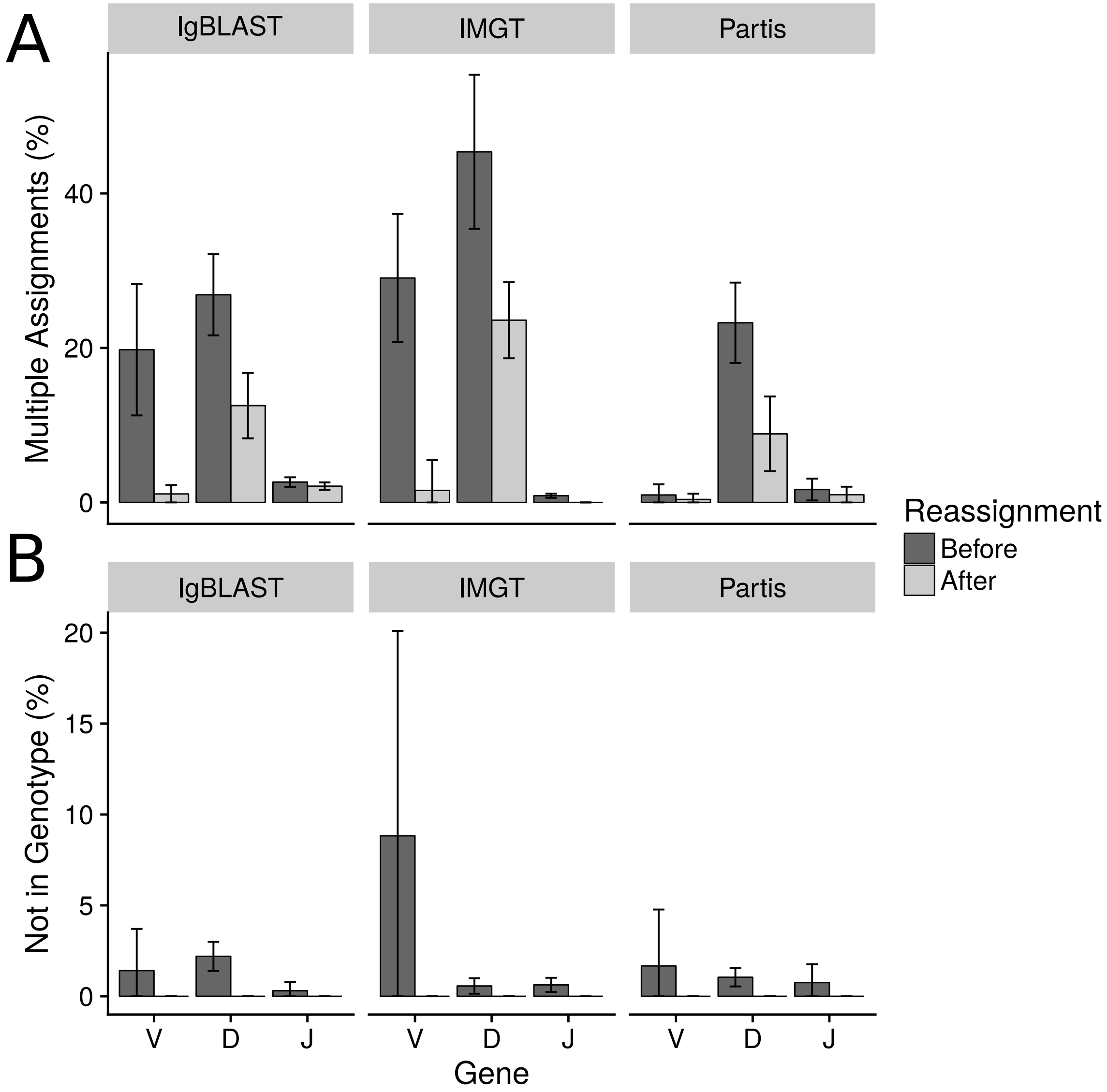

Supplementary Figure 1: **Genotyping reduces multiple allele assignments** The mean percent of multiple assignments and the percentage of genes not in genotype for all individuals across antibody heavy chain genes, before and after genotyping. Alignment was performed using IgBLAST, partis or IMGT. (A) Multiple assignment is reduced by approximately half. (B) After the second assignment, genes originally not assigned within the personal genotype were eliminated. Alignment with partis was applied using version $0.13.0$[1]. In order to obtain the data for the pre-genotype process and the genotype annotation we provided the aligner with our own customized germlines. We used the following flags for partis: '- -skip-unproductive','- -dont-find-new-alleles', and '- -initial-germline-dir'. We ran the received data through an in-house parser to get the needed layout for TIgGER use, there we defined a multiple assignment when the difference in score between the first assignment and the others was less than 0.2. Alignment with IMGT/HighV-QUEST[2] was applied using version $1.5.7.1$ with a parameter modification to allow more assignment in *D* genes. Since IMGT does not accept customized germline, we had to rename the genes after the first alignment. To implement the second realignment step with the individual's own customized reference, we used the reassignAlleles function with Hamming distance from TIgGER for the *J* genes, and a weighted Levenshtein distance for *D* genes.

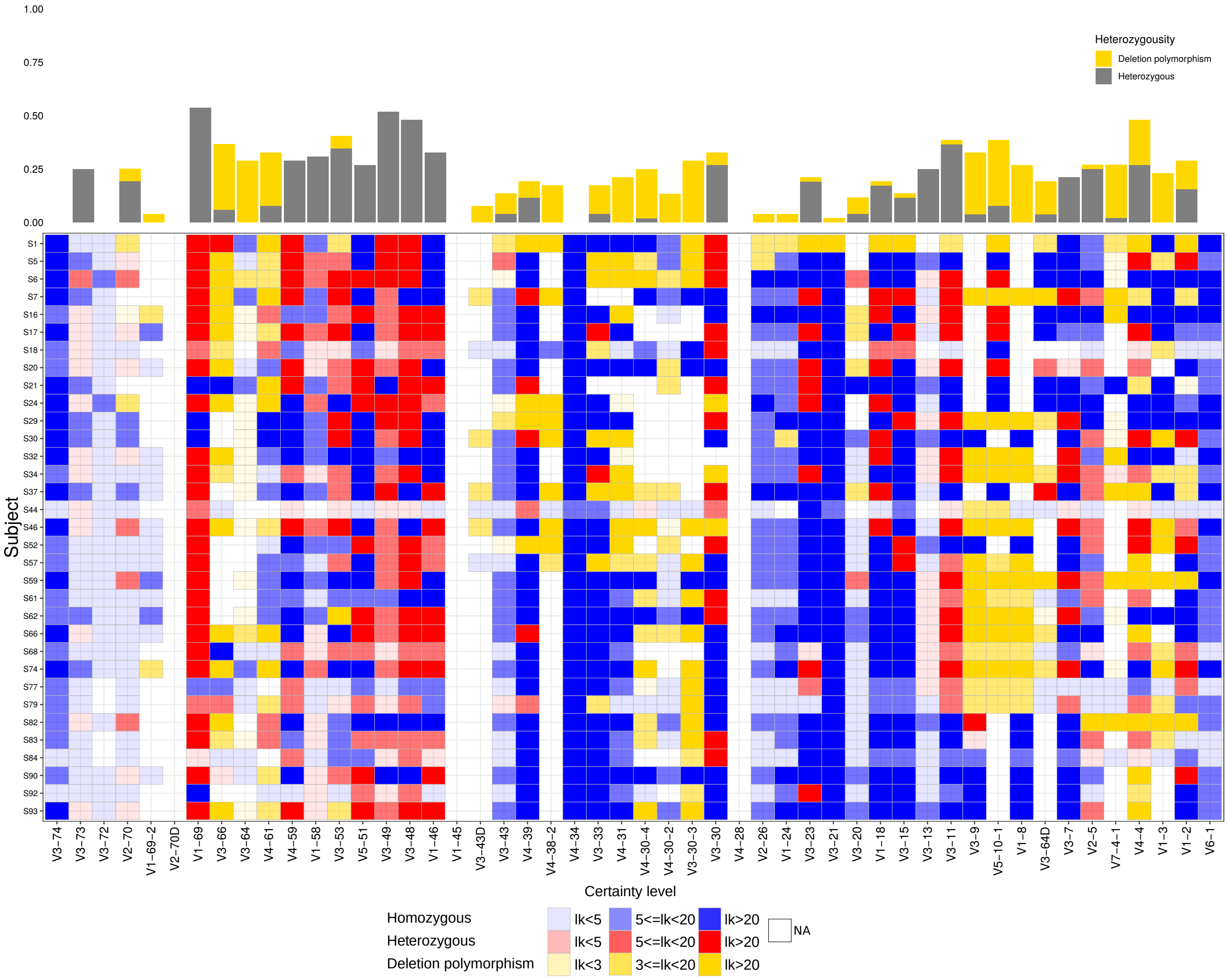

Supplementary Figure 2: **Heterozygosity of the IGH genes including deletion polymorphisms.** Each row represents a *J6* heterozygous individual, and each column represents a *V* gene. Red shades represent heterozygous genes, blue shades homozygous genes, and yellow shades genes with deletion polymorphisms. Transparency corresponds to the certainty level of genotype or haplotype inference. White represents a gene with too low usage (fewer than 10 sequences) to enable clear genotype inference. Bars on top represent the ratio between the number of individuals with heterozygous genes and all individuals with a defined genotype for this gene. Bar color correspond to the type of heterozygosity (deletion polymorphism, and heterozygosity due to two distinct alleles).

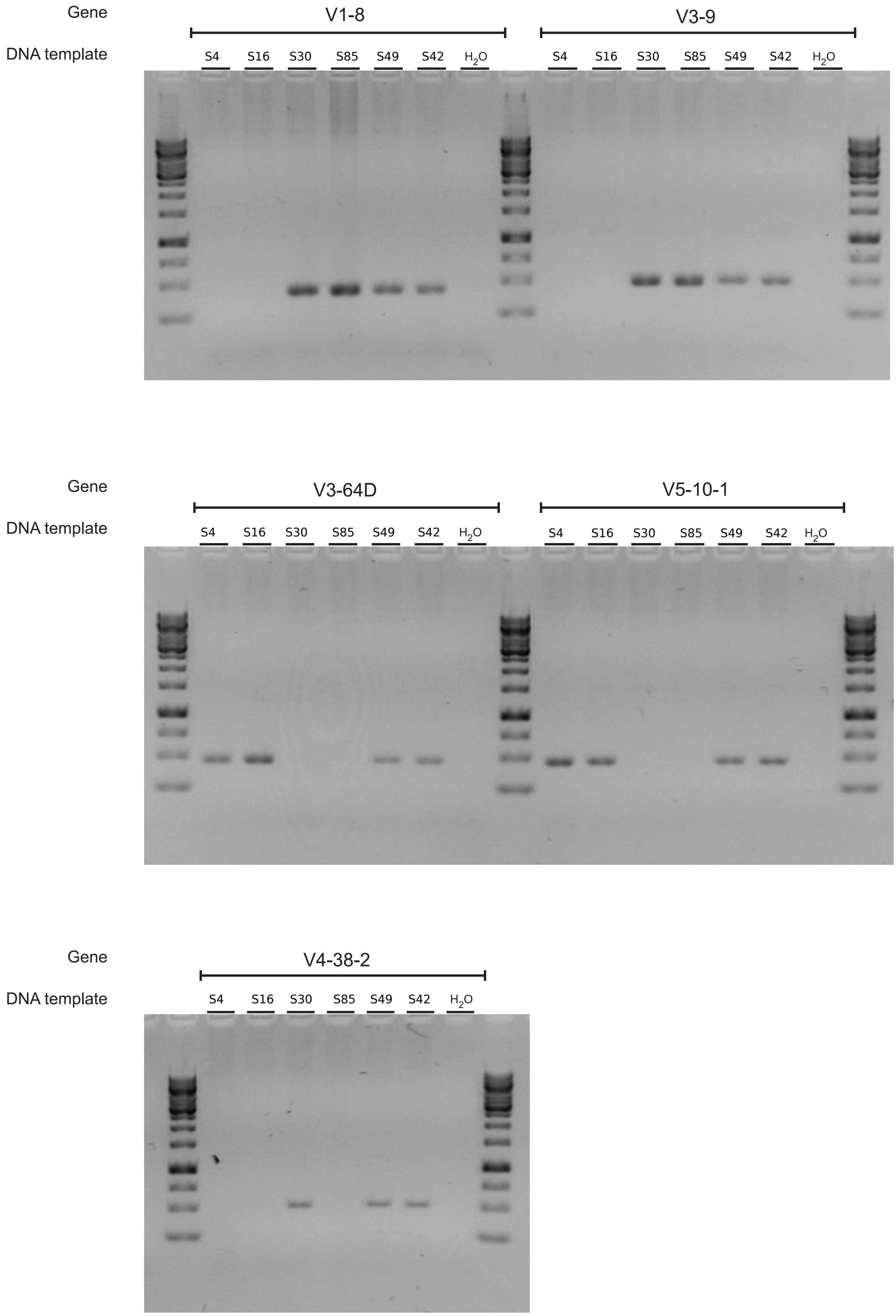

Supplementary Figure 3: **V-gene deletions on the genomic level.** Five genes were amplified by custom-designed gene-specific primers from gDNA and the products were analyzed by gel electrophoresis. GeneRuler 1kb (ThermoFisher) was used as a ladder. As a negative control, water was used instead of a gDNA template. Individuals S42 and S49, who were not predicted to have deletions of the tested genes, were used as positive controls.

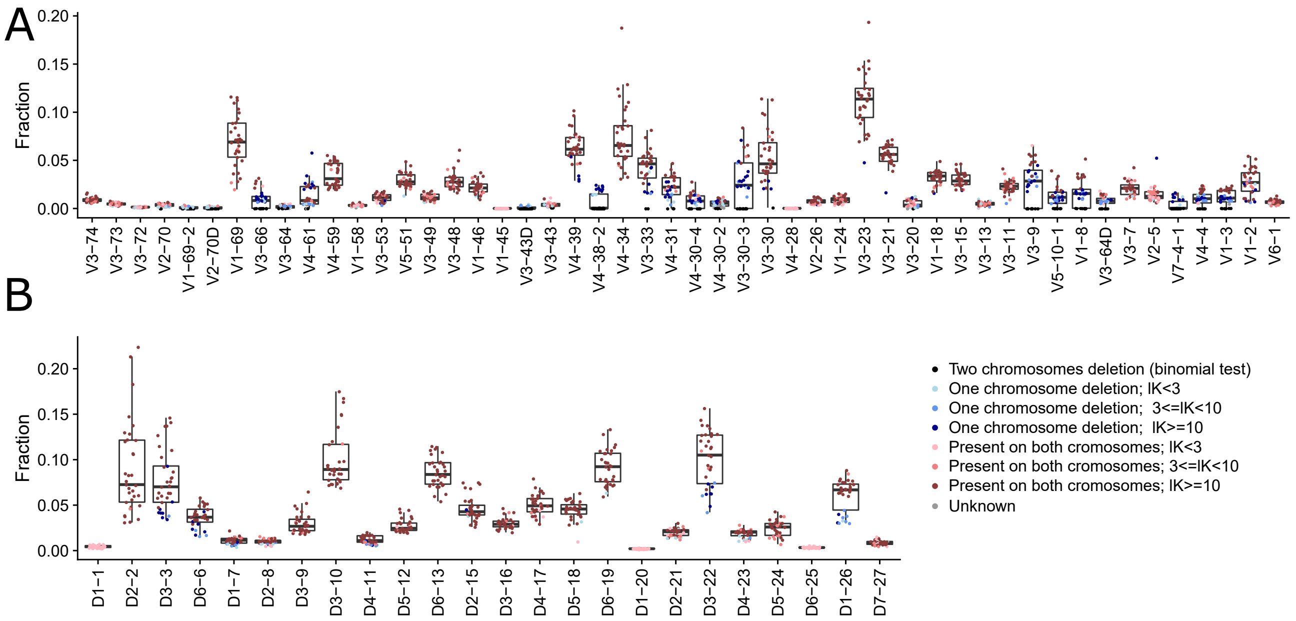

Supplementary Figure 4: **Relative gene usage of *J6* heterozygous individuals.** Relative usage of *V* (A) and *D* (B) genes from *J6* heterozygous individuals. Each dot represents an individual. Color corresponds to gene deletion from both chromosomes (black), single chromosome (blue), or no deletion (red). Shades correspond to the certainty level of deletion inference.

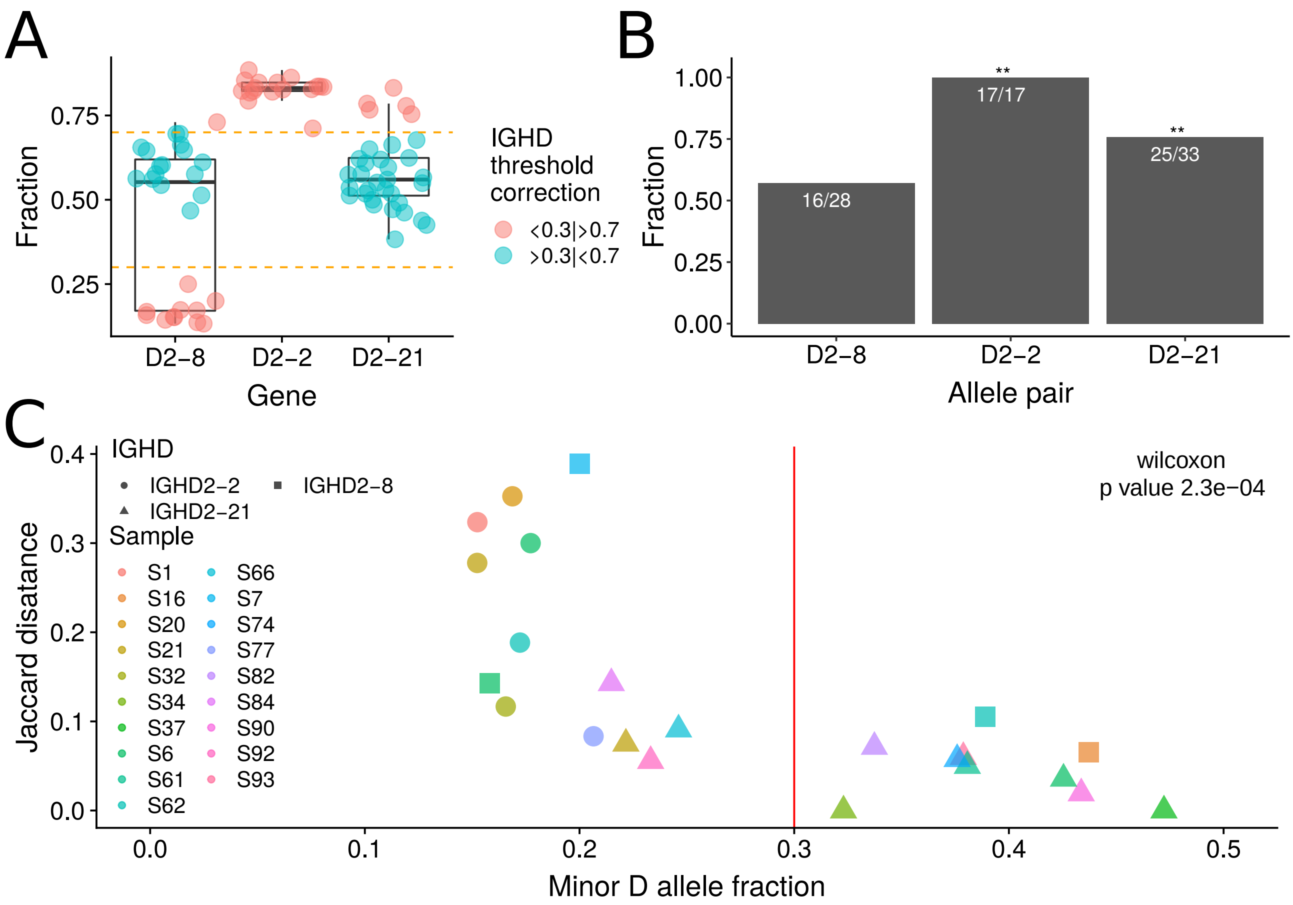

Supplementary Figure 5: ***A D* gene cutoff to be considered as heterozygous** Determining the heterozygosity cutoff for anchor *D* genes. (A) Box plot of anchor *D* gene allele pairs relative usage in heterozygous individuals. We considered allele pairs which were observed in more than 5 individuals, and each individual had a minimum of 5 *V-D-J* linkages for each of the alleles. Each point represents an individual. The allele fraction (Y axis) corresponds to the allele that is written first for each *D* gene, and is the dominant allele in most individuals. (B) The fraction of individuals with relative allele usage (of the first written allele, as in (A)) that is larger than 0.5. Asterisks indicate allele pairs with a statistically significant difference in the number of individuals with the same dominant allele. Statistical significance was determined with a binomial single sample sign test (see methods, $*$ indicates p value < 0.05, $**$ indicates p value < 0.01). (C) To estimate the distance between two haplotypes inferred by different genes, a Jaccard distance was calculated (see methods). The maximum Jaccard distance between the two anchor *D* gene alleles was plotted against the minor allele fraction, where each point represents an individual that had a minimum of 5 genes tested in the Jaccard comparison and each of the genes had a minimum of five linkages with the question allele. This resulted in a reduction in *D* heterozygous samples to 31% for *D2-21* and 17% for *D2-8* (Supplementary Figure 6A). The three shapes of the dots: circle, triangle, and square correspond to the three anchor *D* genes *D2-2, D2-21,* and *D2-8*, respectively. Wilcoxon test was used to assert the cutoff that differentiates between the two groups' means. A cutoff of 0.3 was set with a p-value $< 2e - 03$.

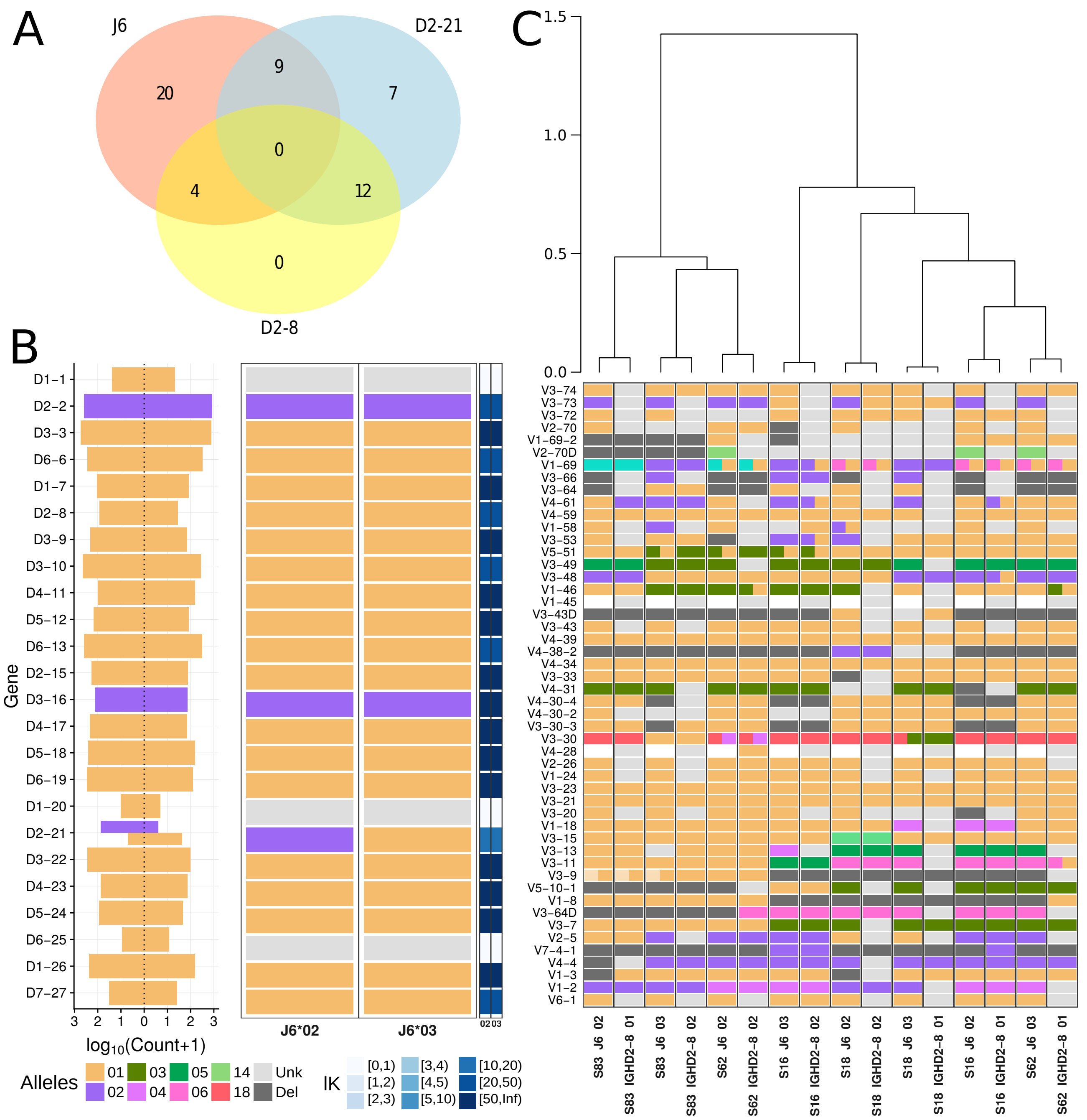

Supplementary Figure 6: ***D*-based haplotype inference.** (A) A Venn diagram of heterozygous individuals for *J6*, *D2-8* and *D2-21* genes. 54% of the individuals were identified as heterozygous in at least one of these anchor genes. (B) An example for *D* gene haplotype anchored by *J6* gene for a single individual. The left panel shows the count of each *D* gene (Y axis) that is associated with its paired anchor *J6* gene (X axis). The thickness of the count bar is inversely proportional to the number of alleles found on the chromosome. Colors correspond to the different alleles. The middle panel shows the called *D* haplotype, and the right panel shows the certainty level (lK) for each haplotype decision. (C) V haplotype map where each column corresponds to a *J6*- or *D2-8*-based haplotype of an individual that is heterozygous for both these genes. The order of columns was determined by a hierarchical dendrogram based on the distances between individual haplotypes.

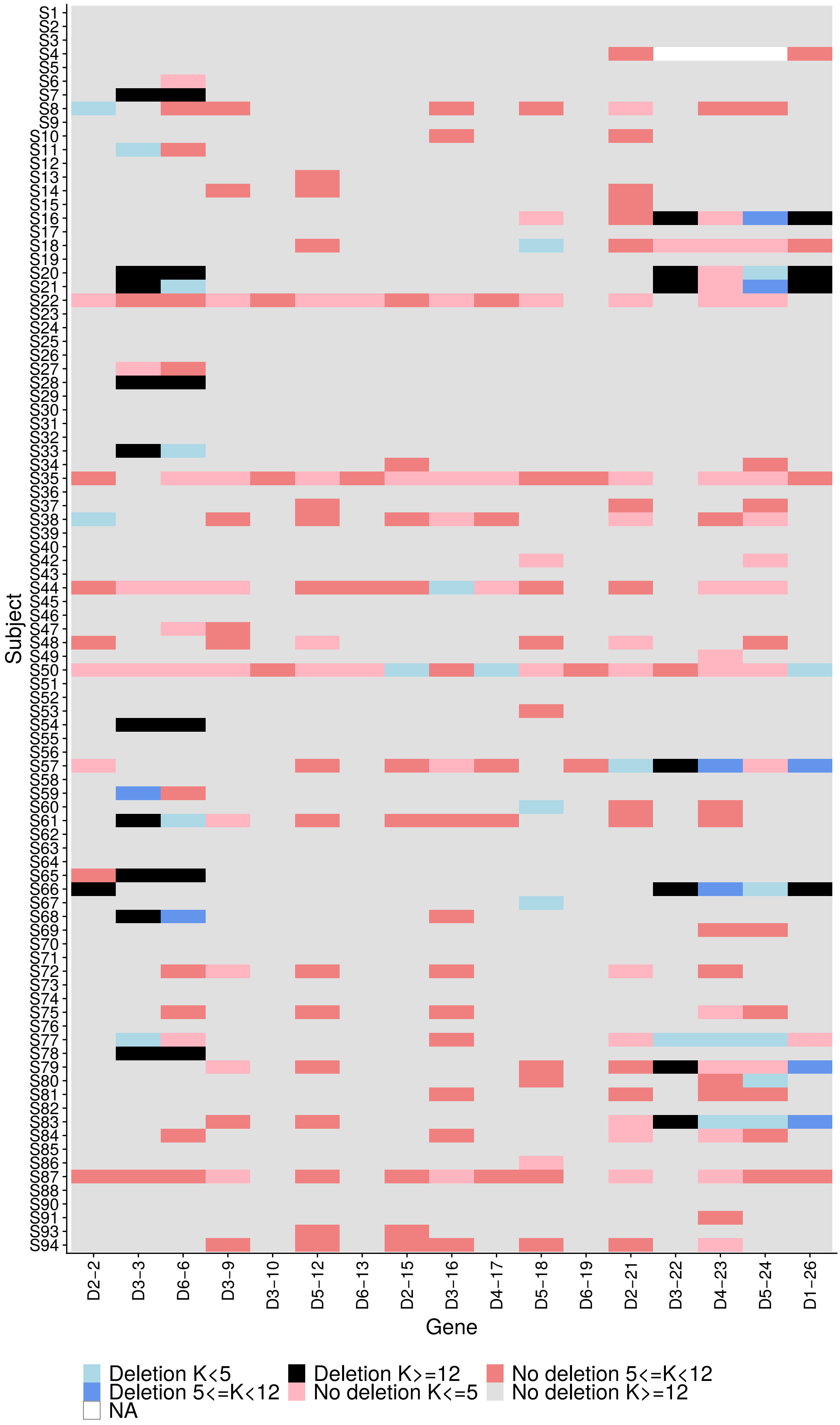

Supplementary Figure 7: **Chromosome deletion detection by pooled heterozygous *V* genes in the entire cohort.** For each individual (Y axis) and gene (X axis), each cell represents the deletion status of a gene (deletion/no deletion). The color indicates the certainty level of the decision. For the presented V_{pooled} approach only heterozygous V genes with minor allele fraction larger than 30% were included.

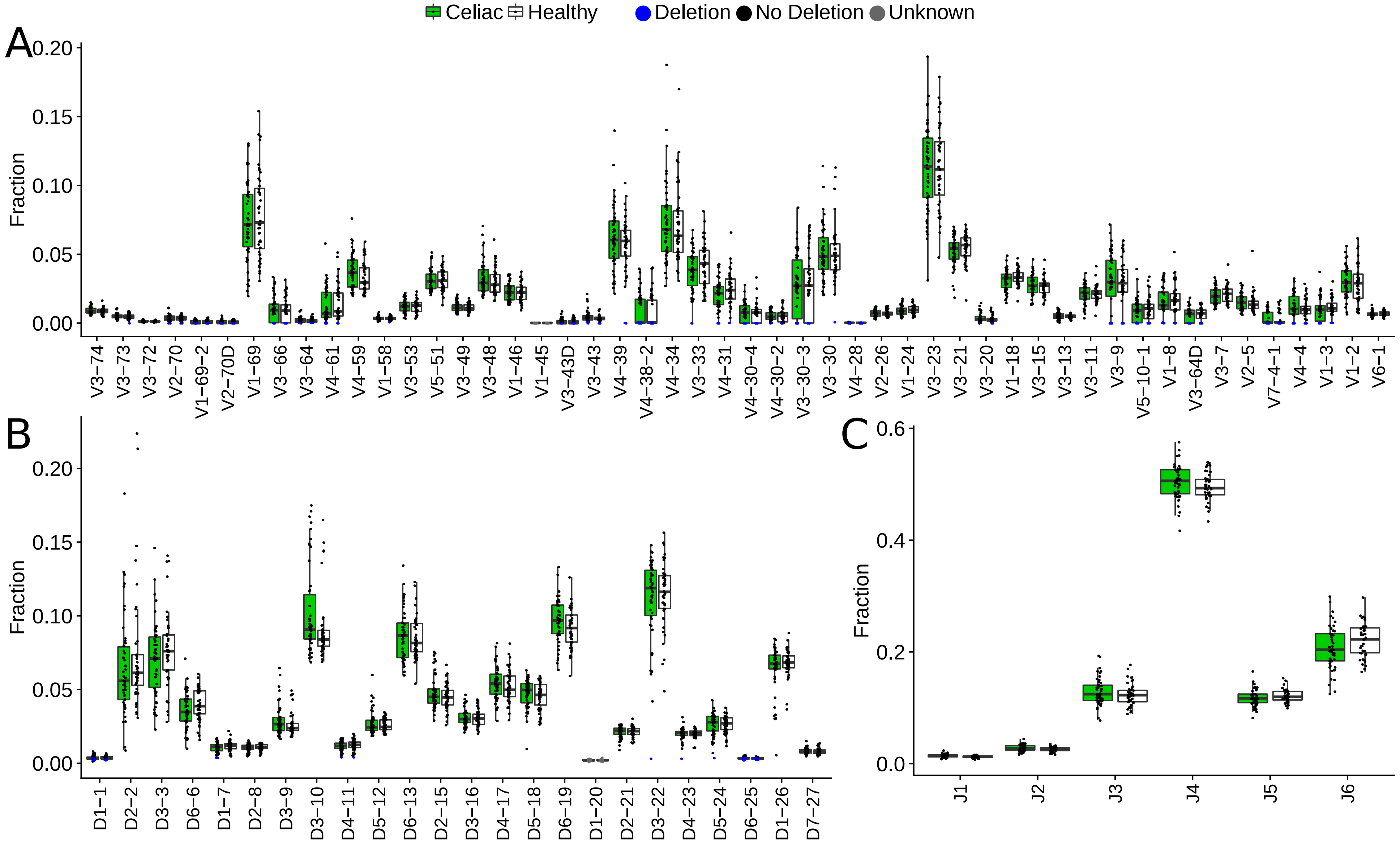

Supplementary Figure 8: **Gene deletion inference by relative gene usage with respect to clinical status.** Box plots of relative gene usage, where each dot represents a single individual, separated into celiac patients (green) and healthy individuals (white), for *V* (panel A), *D* (panel B), and *J* (panel C) genes. Blue represents deleted genes according to the binomial test (see methods).

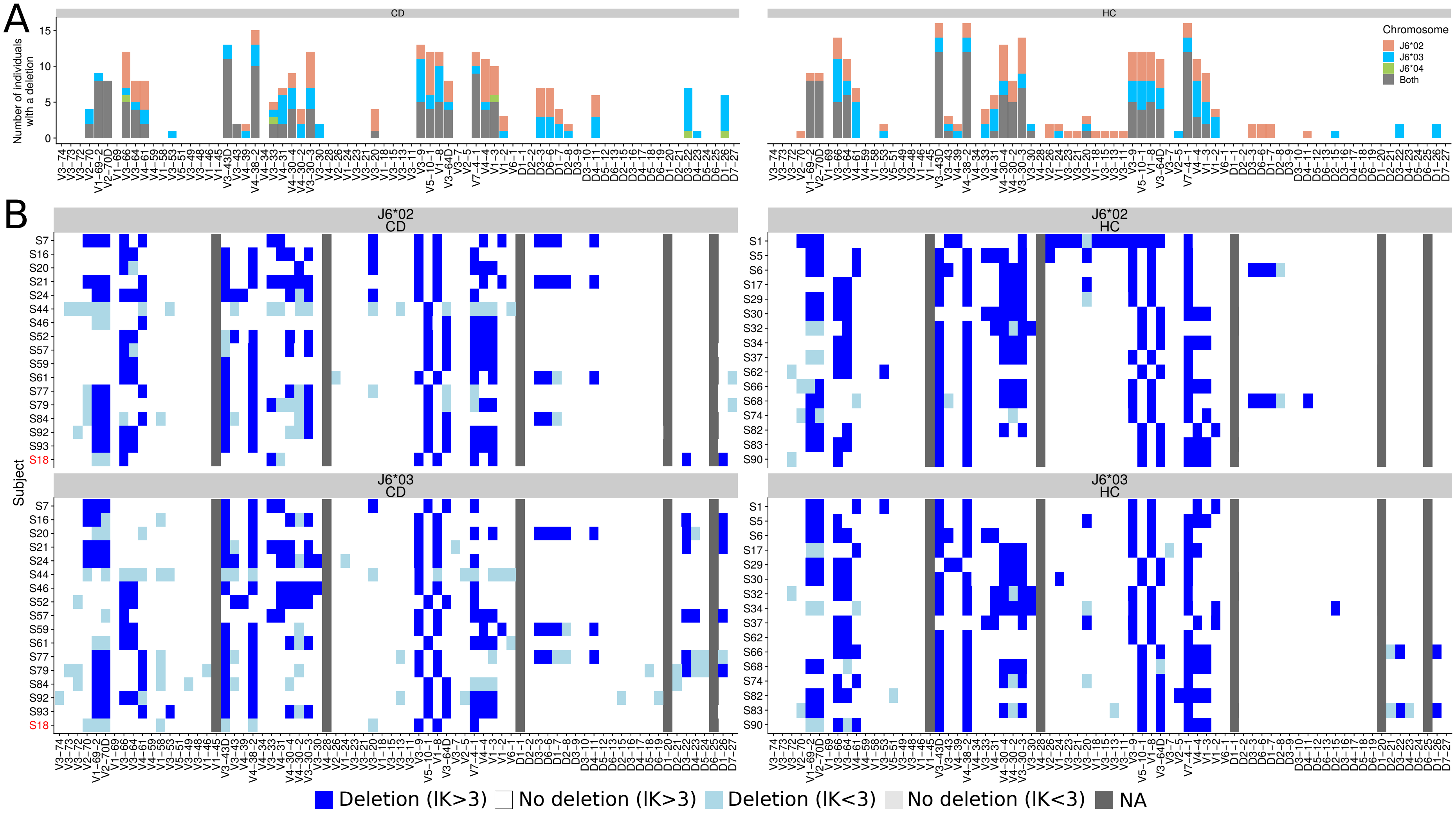

Supplementary Figure 9: **Gene deletion inference along each chromosome according to clinical status.** (A) The distribution of V and D gene deletions along each chromosome in 32 individuals that are heterozygous for *J6*, separated into celiac disease patients (left panel) and healthy individuals (right panel), as inferred by haplotype (light red, blue, and green) and by the binomial test (gray) (B) A heatmap of V and D gene deletions and suspected deletions for each of the 32 heterozygous individuals in *J6*, separated into celiac patients (left panel) and healthy individuals (right panel). Each row represents an individual, and each column represents V or D gene. Blue represents a deletion ($lK > 3$), light blue represents a suspected deletion ($lK < 3$), and light gray represents no deletion on both chromosomes with low certainty ($lK < 3$). Dark gray represents a gene with an extremely low usage across all samples. The top panel represents the chromosome on which *J6*02* is present, and the bottom panel represents the chromosome on which *J6*03* is present. Sample S18, marked in red, is heterozygous for *J6*03* and *J6*04*. For this individual, *J6*04* was added to the *J6*02* panel.

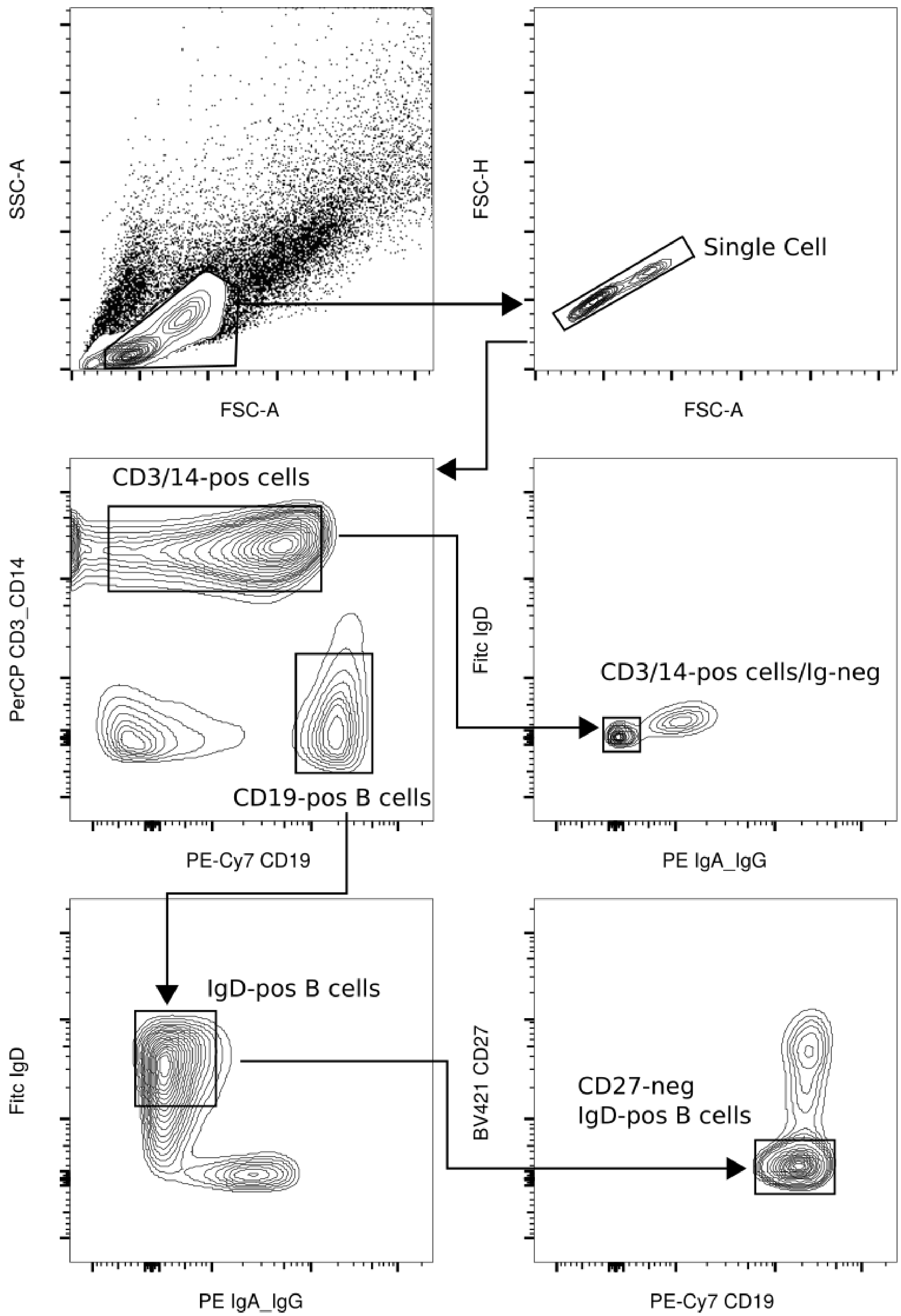

Supplementary Figure 10: **Gating strategy for cell sorting of non B cells and naïve IgD+ B cells.** Gating strategy for sorting non-B cells (for gDNA extraction) and naïve IgD-expressing B cells (for RNA extraction). Non-B cells were sorted for being CD19-negative, CD3/CD14-positive and negative for any true/false signal from anti-IgD/IgA/IgG antibodies (mid right panel). Naïve B cells were defined as CD19-positive cells that were negative for CD3 and CD14, IgD-positive and IgA/IgG-negative that were also negative for the memory B marker CD27 (lower right panel). Arrows indicate sequential gating.

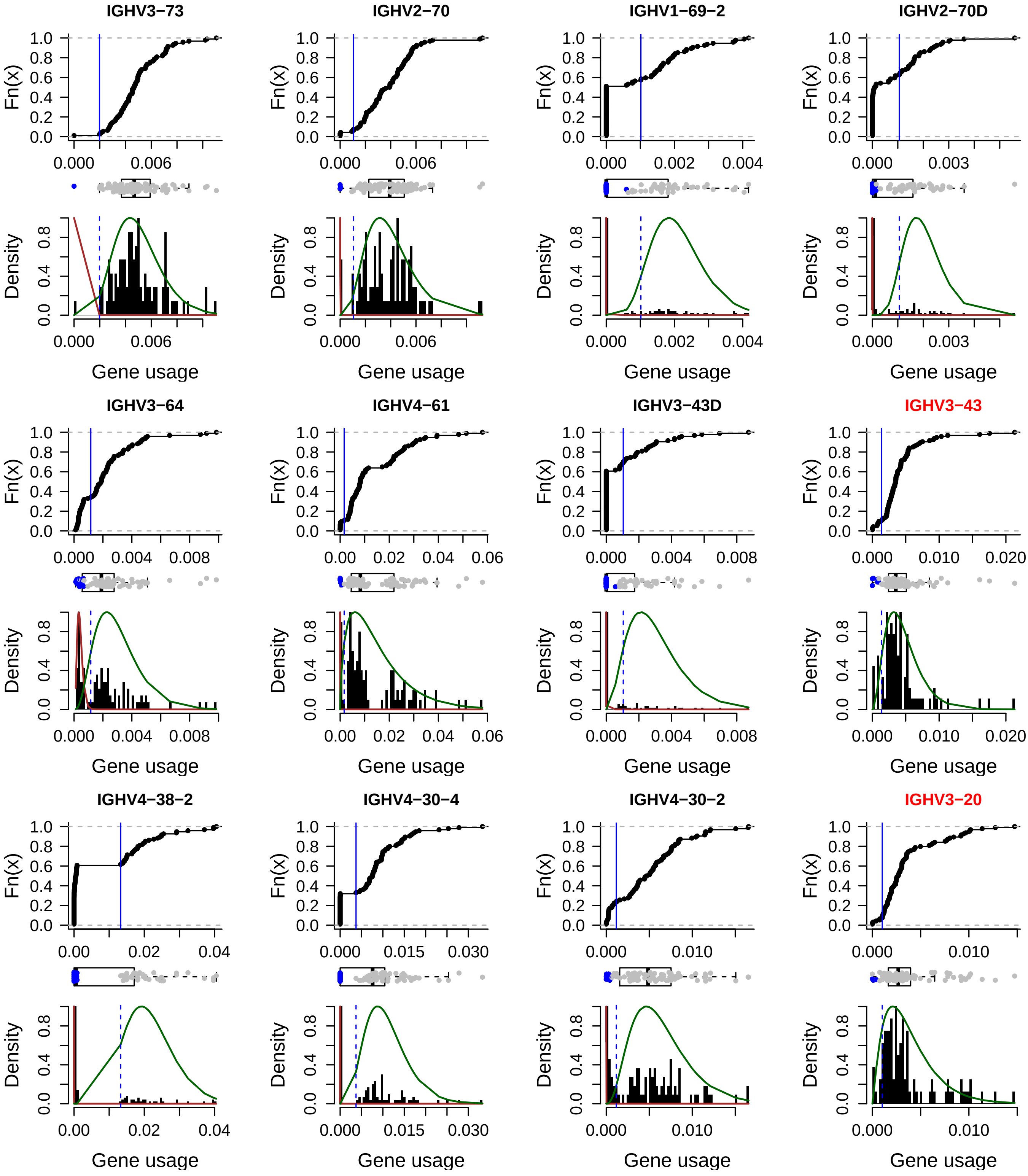

Supplementary Figure 11: **Bimodal distribution of low expressed genes.** For each gene with low usage, an empirical cumulative distribution function curve of the gene usage (upper panel for each gene), boxplot of the gene usage where the deletions are marked in blue (middle panel for each gene), and a histogram of the gene usage with the estimated gamma distributions(lower panel for each gene) are shown. Genes with labels marked in red do not follow a bimodal behavior, therefore deletion detection is considered less reliable for them.

Supplementary Tables

Sample	Number of naive B cells	RIN	Paired sequences	Unique sequences	Single assignment VDJ
S1	3.5e5	10	936044	34756	28977
S2	4.5e5	9.8	896721	31108	26849
S3	4.2e5	9.4	736291	38716	32937
S4	3.5e5	8.8	872281	26747	21282
S5	3.5e5	8.5	662652	37984	32223
S6	4e5	9.5	1016502	53829	43847
S7	3e5	9.4	717247	42842	36776
S8	3.5e5	9.3	821769	5469	4464
S9	7e5	8.5	747468	34650	29217
S10	2.5e5	7.6	953488	6656	5595
S11	3.5e5	9.1	847040	26612	22682
S12	3.5e5	9.9	788419	30277	25721
S13	3e5	9.3	917742	54470	45287
S14	3.5e5	10	625901	19042	16499
S15	2.4e5	8.5	1006142	54030	45021
S16	2.5e5	9.2	876395	39773	33357
S17	3e5	7	1192473	26036	21957
S18	8.5e5	8	1206532	7798	6218
S19	4e5	9.1	781842	46688	39487
S20	5e5	8.7	537152	27361	22607
S21	3.5e5	9.7	1133385	27712	22205
S22	3.5e5	8.5	815183	11902	10170
S23	2.8e5	9.3	967969	48795	42069
S24	4e5	8.9	700587	35643	29149
S25	4.5e5	8.8	749075	34025	28290
S26	2.5e5	9.5	1115341	55694	46951
S27	2.3e5	9.6	716100	33553	28048
S28	1e5	9.2	775067	28194	23614
S29	3.5e5	8.6	841336	37040	31401
S30	3.5e5	9.6	1044881	37573	32322
S31	6e5	8.8	786272	36442	30946
S32	2.5e5	9.7	653913	22147	18532

Supplementary Table 1: **Sequencing data summary.** The first column represents the sample name. The second column shows the number of sorted naïve B cells. The third column shows the sample RIN scores. The fourth column shows the number of assembled paired sequences for each sample. The fifth column shows the number of sequences after the initial *VDJ* alignment using IgBLAST, which were functional, had a read count above 2, a *V* gene mutation count below 3, and no mutations in the *D* gene. The sixth and last column shows the number of sequences with a single assignment for each of the *VDJ* genes for each sample.

Sample	Number of naive B cells	RIN	Paired sequences	Unique sequences	Single assignment VDJ
S33	3.5e5	8.6	820166	27688	23960
S34	2.5e5	8.1	699674	18231	15428
S35	2.5e5	7.8	886829	6270	5439
S36	3.5e5	9.2	777346	36919	31690
S37	4e5	9	796176	39383	33301
S38	2.5e5	7.2	646166	4074	3471
S39	5e5	9.8	678202	19349	15486
S40	2.5e5	7.8	948734	12746	10942
S41	1.5e5	7.2	780665	2548	2109
S42	3.5e5	9.1	717585	30336	25666
S43	5e5	8.5	582384	36305	30872
S44	3e5	7.5	730609	3117	2708
S45	1.1e5	9.3	814191	17652	15363
S46	3.5e5	9.9	933332	39848	33827
S47	2.5e5	10	1014845	32405	27696
S48	3e5	6.4	1021241	5756	4870
S49	3.8e5	9.3	634033	14603	12470
S50	3e5	8.5	777167	15824	13442
S51	3e5	8.9	789479	27543	23722
S52	5e5	8.7	880247	31001	26093
S53	1.2e5	9.1	974061	22964	19558
S54	4e5	9	989353	50241	44485
S55	2.5e5	9.1	946647	39230	33253
S56	4.5e5	8.7	903587	41298	35776
S57	3.5e5	8.4	756901	13783	12083
S58	9e5	6.8	618095	35802	30762
S59	2.5e5	9.3	906100	36518	32093
S60	2.8e5	8.8	974636	45215	38689
S61	2.6e5	8.8	473494	10419	9098
S62	1.8e5	9	804185	35565	29070

Supplementary Table 1: **Sequencing data summary (Cont.)**

Sample	Number of naive B cells	RIN	Paired sequences	Unique sequences	Single assignment VDJ
S63	2.5e5	9.4	892123	28823	23674
S64	3.5e5	8.8	1247072	50146	43362
S65	2.5e5	9.4	757705	37277	31002
S66	4e5	9.3	1089824	46583	40108
S67	2.8e5	9.7	816466	32359	27960
S68	3e5	9.1	841563	9949	8559
S69	4e5	9.1	952996	51314	44066
S70	4e5	7.9	711166	23258	20097
S71	4e5	8.6	979793	28455	24291
S72	3.1e5	8.4	741015	6594	5732
S73	4e5	8.2	679834	35316	30945
S74	4e5	9	737264	38276	32565
S75	2.8e5	9.3	977269	13592	11917
S76	3e5	8.9	829329	46567	39337
S77	3.5e5	9.8	1150471	6281	5117
S78	3e5	7.3	1002758	13714	12056
S79	3.5e5	9	917183	7880	6642
S80	1.1e5	9.8	576024	15714	13135
S81	1.2e5	9.4	795323	27966	23910
S82	4e5	9.2	677994	32794	28225
S83	3e5	9	927048	11647	8634
S84	3e5	9.2	569276	8066	6678
S85	3e5	8.8	900189	11158	9440
S86	2.5e5	8.4	745469	21820	18232
S87	3.5e5	9.6	1456927	7558	6530
S88	1.5e5	9.2	902418	36937	31043
S89	4e5	8.9	666271	34469	29569
S90	3.5e5	9.3	915147	28631	24805
S91	2.3e5	9.4	823833	26501	22922
S92	2.5e5	8.8	1386237	8624	7278
S93	2.5e5	7.7	730300	29955	25341
S94	4e5	8.7	493745	21285	18253

Supplementary Table 1: **Sequencing data summary (Cont.)**

A

	10^6 Cells	ng/µl
S4	3.5	46.4
S16	3.0	38.7
S30	4.3	57.1
S85	3.5	57.5
S49	3.5	46.8
S42	3.0	50.9

B

	Sequence (5'-3')
IGHV1-8_fwd	GTAAGGGGCTTCCTAGTCTCAAAG
IGHV1-8_rev	TCTGACTCTCTGAGGATGTGGTTT
IGHV3-9_fwd	AGGACTCACCATGGAGTTGG
IGHV3-9_rev	TTTTTGTCTGGGCTCTCGCT
IGHV3-64D_fwd	TTCATGGAGAACTAGAGATAGTGTG
IGHV3-64D_rev	GCTGTTTTTCTCCAGCGTTCC
IGHV4-38-2_fwd	AGGGATCCAGACGTGAAGATA
IGHV4-38-2_rev	GGCCTTGTATTCCGTGAGC
IGHV5-10-1_fwd	CATCCTTGGCCTCCTCCTG
IGHV5-10-1_rev	GGGTTTTAGACGGGCTCAGT

Supplementary Table 2: **Amplification of selected immunoglobulin variable heavy chain genes from gDNA.** (A) Cell number and gDNA concentration from T cells and monocytes together (non-B cells). (B) Custom-designed primers for amplification of selected immunoglobulin variable heavy chain genes from gDNA .

SUBJECT−gene−primer	IMGT annotation	Identity	Mutation
S42−HV1−8−HV1−8_fwd	Homsap IGHV1−8*01 F	100.00%.(288/288 nt)	−
S42−HV3−9−HV3−9_fwd	Homsap IGHV3−9*01 F	100.00%.(288/288 nt)	−
S42−HV3−64D−HV3−64D_fwd	Homsap IGHV3−64D*06 F	99.65%.(287/288 nt)	g258>t (T86)
S42−HV4−38−2−HV4−38−2_fwd	Homsap IGHV4−38−2*01 F	100.00%.(288/288 nt)	−
S42−HV5−10−1−HV5−10−1_fwd	Homsap IGHV5−10−1*03 F	100.00%.(288/288 nt)	−
S49−HV1−8−HV1−8_fwd	Homsap IGHV1−8*01 F	100.00%.(288/288 nt)	−
S49−HV3−9−HV3−9_fwd	Homsap IGHV3−9*01 F	100.00%.(288/288 nt)	−
S49−HV3−64D−HV3−64D_fwd	Homsap IGHV3−64D*06 F	99.65%.(287/288 nt)	g258>t (T86)
S49−HV4−38−2−HV4−38−2_fwd	Homsap IGHV4−38−2*01 F	100.00%.(288/288 nt)	−
S49−HV5−10−1−HV5−10−1_fwd	Homsap IGHV5−10−1*03 F	100.00%.(288/288 nt)	−
S85−HV1−8−HV1−8_fwd	Homsap IGHV1−8*01 F	100.00%.(288/288 nt)	−
S85−HV3−9−HV3−9_fwd	Homsap IGHV3−9*01 F	100.00%.(288/288 nt)	−
S30−HV1−8−HV1−8_fwd	Homsap IGHV1−8*01 F	100.00%.(288/288 nt)	−
S30−HV3−9−HV3−9_fwd	Homsap IGHV3−9*01 F	100.00%.(288/288 nt)	−
S30−HV4−38−2−HV4−38−2_fwd	Homsap IGHV4−38−2*02 F	100.00%.(288/288 nt)	−
S16−HV3−64D−HV3−64D_fwd	Homsap IGHV3−64D*06 F	100.00%.(288/288 nt)	−
S16−HV5−10−1−HV5−10−1_fwd	Homsap IGHV5−10−1*01 F	100.00%.(288/288 nt)	−
S4−HV3−64D−HV3−64D_fwd	Homsap IGHV3−64D*06 F	100.00%.(288/288 nt)	−
S4−HV5−10−1−HV5−10−1_fwd	Homsap IGHV5−10−1*03 F	100.00%.(288/288 nt)	−

Supplementary Table 3: **Sanger sequencing of PCR products.** PCR products, obtained by amplification of genomic DNA of selected individuals using gene-specific primers, were sequenced to confirm the specificity of the primers and verify the identity of the targeted genes. Novel allele *IGHV3-64D*06_G258T* was also found by TIgGER as part of 25 novel alleles described in methods.

Gene	mu1	sd1	n1	mu2	sd2	n2	Sensitivity 0.01	Threshold 0.01	Sensitivity 0.05	Threshold 0.05
V3−66	0.0109	0.0026	14	0.0270	0.0043	4	0.1007	0.0072	0.9786	0.0165
V3−53	0.0071	0.0018	2	0.0123	0.0034	18	0.1469	0.0033	0.3633	0.0060
V4−39	0.0369	0.0116	4	0.0683	0.0134	25	0.4355	0.0348	0.7393	0.0453
V3−33	0.0271	0.0126	6	0.0492	0.0125	22	0.2443	0.0174	0.5175	0.0276
V4−31	0.0196	0.0063	8	0.0326	0.0070	13	0.1921	0.0135	0.5264	0.0198
V4−30−4	0.0093	0.0026	7	0.0171	0.0058	6	0.0062	0.0000	0.0989	0.0054
V4−30−2	0.0075	0.0014	4	0.0089	0.0019	5	0.0127	0.0018	0.0761	0.0048
V3−30−3	0.0339	0.0148	11	0.0633	0.0138	5	0.0824	0.0114	0.5009	0.0336
V3−30	0.0235	0.0046	3	0.0574	0.0256	27	0.0184	0.0000	0.0852	0.0135
V2−26	0.0055	0.0017	2	0.0087	0.0019	21	0.2511	0.0036	0.4709	0.0051
V3−20	0.0103	0.0015	2	0.0082	0.0026	4	0.0469	0.0000	0.0586	0.0018
V3−9	0.0305	0.0083	11	0.0482	0.0083	6	0.1253	0.0201	0.5486	0.0315
V5−10−1	0.0120	0.0036	10	0.0238	0.0089	9	0.0042	0.0000	0.1088	0.0072
V1−8	0.0156	0.0029	11	0.0344	0.0092	7	0.0035	0.0054	0.6331	0.0165
V3−64D	0.0084	0.0016	3	0.0138	0.0023	4	0.0462	0.0033	0.5042	0.0084
V4−4	0.0104	0.0013	10	0.0201	0.0030	7	0.6163	0.0105	0.9920	0.0141
V1−3	0.0102	0.0017	8	0.0201	0.0019	11	0.9831	0.0147	0.9960	0.0165
V1−2	0.0157	0.0079	5	0.0321	0.0144	20	0.0592	0.0000	0.1718	0.0072
D3−3	0.0486	0.0189	8	0.0860	0.0299	23	0.0432	0.0108	0.2420	0.0345
D6−6	0.0278	0.0092	6	0.0421	0.0083	23	0.2535	0.0210	0.4996	0.0276
D4−11	0.0086	0.0034	2	0.0137	0.0031	21	0.2901	0.0057	0.4828	0.0081
D3−22	0.0621	0.0088	5	0.1165	0.0219	22	0.4730	0.0613	0.9363	0.0787
D1−26	0.0355	0.0065	2	0.0706	0.0067	23	0.8908	0.0538	0.9139	0.0589

Supplementary Table 4: **Threshold statistics for figure 6.** The annotation $mu1, sd1, n1$ represent the single chromosome deletion group, and $mu2, sd2, n2$ for no deletion.

Supplementary References

[1] Ralph, D. K. & Matsen IV, F. A. Consistency of VDJ rearrangement and substitution parameters enables accurate B cell receptor sequence annotation. *PLoS computational biology* **12**, e1004409 (2016).

[2] Li, S. *et al.* IMGT/HighV QUEST paradigm for T cell receptor IMGT clonotype diversity and next generation repertoire immunoprofiling. *Nature communications* **4**, 2333 (2013).
